# Fatty Acid Profile, Health Lipid Indices, and Sensory Properties of Meat from Pekin Ducks of Different Origins

**DOI:** 10.3390/ani13132066

**Published:** 2023-06-22

**Authors:** Rafał Wasilewski, Dariusz Kokoszyński, Karol Włodarczyk

**Affiliations:** Department of Animal Breeding and Nutrition, Faculty of Animal Breeding and Biology, Bydgoszcz University of Science and Technology, 85084 Bydgoszcz, Poland

**Keywords:** duck, Pekin, genetic resource, meat, fatty acid profile, sensory trait

## Abstract

**Simple Summary:**

Native or locally adapted poultry breeds are characterized by good adaptation to local environmental conditions. They are characterized by a unique genotype, high resistance to existing diseases, and very good reproductive ability, as well as good-quality raw material. The aim of the present study was to compare P33 (Polish Pekin), P8 (Danish Pekin), and P9 (French Pekin) ducks at 49 days of age in terms of fatty acid profile, health lipid indices, and sensory characteristics of breast and leg meat. The results showed significant differences between the compared Pekin duck strains in terms of the content of certain fatty acids and sensory characteristics, which may indicate the different nutritional and culinary values of the meat of the compared conservative duck strains.

**Abstract:**

Conservation duck flocks are of significant importance to science. Over a number of years, many experiments have been carried out to gain a better understanding of individual duck populations. However, the knowledge obtained is still incomplete. The aim of the present study was to compare three duck strains maintained in Poland and included in the Genetic Resources Protection Programme in terms of the fatty acid profile, health lipid indices, and sensory traits of breast and leg meat. The experimental material consisted of 180 sexed Pekin ducks, 60 ducks each (including 30 males and 30 females) from strain P33 (Pekin of Polish origin), P8 (Pekin of Danish origin), and P9 (Pekin of French origin). During 49 days of rearing, the ducks were kept in an enclosed building with six pens on straw. The duck genotype had a significant effect on the myristic (C14:0), palmitic (C16:0), oleic (C18:1n9), linolenic (C18:2n6), arachidonic (C20:4n6), monounsaturated fatty acid (MUFA), PUFAn6 content, and Peroxidisability Index (PI) values of breast muscle. Ducks from the compared strains differed significantly in C16:0 content and the proportion of unsaturated fatty acids (UFAs), including MUFAs, Nutritive Value Index, and Health-Promoting Index in leg muscles. Ducks from the compared strains also differed significantly in the aroma and juiciness of the heat-treated breast muscles and the tenderness of the leg muscles. The sex of the birds had a significant effect on the C18:1, C22:6n3, MUFA, PUFAn6, PUFAn3, PI, and aroma and taste desirability of the breast muscles, as well as the aroma intensity of the leg muscles.

## 1. Introduction

Poultry meat, including duck meat, can be considered a food item of high nutritional value due to its higher unsaturated and lower saturated fatty acid content compared to pork and beef. The fatty acid (FA) profile of meat depends on the bird species [1], but can vary significantly depending on the type and amount of fats contained in the feed [2,3,4,5,6]. The FA profile of meat is also influenced by the type of meat [7,8,9], housing system [10,11,12], age [9,13], and sex of the birds [14]. Fatty acids play an important biological role, mainly as energy and storage material. They take part in maintaining cell membrane integrity and chemical transmission. PUFAs affect the maturation of the central nervous system, brain function, reduce the risk of heart attacks, obesity, and some cancers [15,16].

For meat consumers, sensory quality is increasingly important. In the sensory evaluation of meat, the most important attributes are colour, tastiness, tenderness, and juiciness. Tastiness is a characteristic that is a combination of two sensory impressions, i.e., taste and smell. Determining the smell and taste of meat is largely subjective. It depends mainly on the degree of sensory sensitivity of the assessor. Local culinary preferences also influence the score obtained. The precursors of meat taste are mainly glutamic acid and sulphur amino acids as well as serine, lysine, and isoleucine [17,18]. Approximately 500 chemical compounds are responsible for the aroma of poultry meat; these are low-molecular-weight volatile compounds, such as aldehydes, hydrocarbons, ketones, alcohols, esters, and carboxylic acid, derived from lipid degradation [19,20,21]. Most of these are formed during the maturation of meat. According to Fu et al. [21], the hexanal and nonanal aldehydes formed during the oxidation of unsaturated fatty acids have the greatest impact on meat odour. Mature meat is characterised by a more intense odour and taste than meat obtained just after the bird has been slaughtered. The taste and odour of meat are also influenced by the species or breed of poultry, the age of the birds, the type of meat, the acidity of the meat, the nutrition of the birds, and the storage time, among other factors [18,21]. Meat from older birds has a more intense and typical taste and smell compared to meat from younger birds. 

An important sensory attribute of meat is its juiciness. The perception of meat succulence in sensory assessment is influenced, in addition to the amount of juice released during biting and chewing as well as the method and time of thermal treatment before assessment, by factors such as the tenderness of the meat, the degree of fat cover, the correctness of the maturation process of the meat, and the course of thermal treatment [18]. For these reasons, the results of the instrumental assessment of meat juiciness are not always consistent with organoleptic assessments.

Another sensory quality characteristic that determines the culinary quality of meat is its tenderness. It can be assessed instrumentally or organoleptically. Meat tenderness is determined by the type and diameter of the muscle fibres, the proportion of perimysium and epimysium, the rate of proteolytic changes post mortem, the water holding capacity, and the ultimate pH [22,23,24,25]. The meat of young broiler-type commercial birds is more tender than that of older birds due to the smaller diameter of the muscle fibres and the better solubility of the collagen. 

So far, few scientific studies have compared the fatty acid profile and sensory traits of Pekin ducks from the P33, P8, and P9 strains included in the Genetic Resources Protection Programme in Poland, which was the reason for conducting the present study. The aim of this study was to compare 49-day-old genetic resource ducks from the P33 (Polish Pekin), P8 (Danish Pekin), and P9 (French Pekin) strains in terms of the fatty acid profiles, health lipid indices, and sensory traits of breast and leg meat. This study provides information on the differences in the fatty acid profiles, health lipid indices, and culinary suitability of the meat of the compared duck strains, which has important implications for duck meat consumers.

## 2. Materials and Methods

The experiment was conducted with the approval of the Local Ethical Committee for Animal Research in Bydgoszcz—Resolution 21/2014 of 26 June 2014.

### 2.1. Birds and Housing

**Figure 1:**
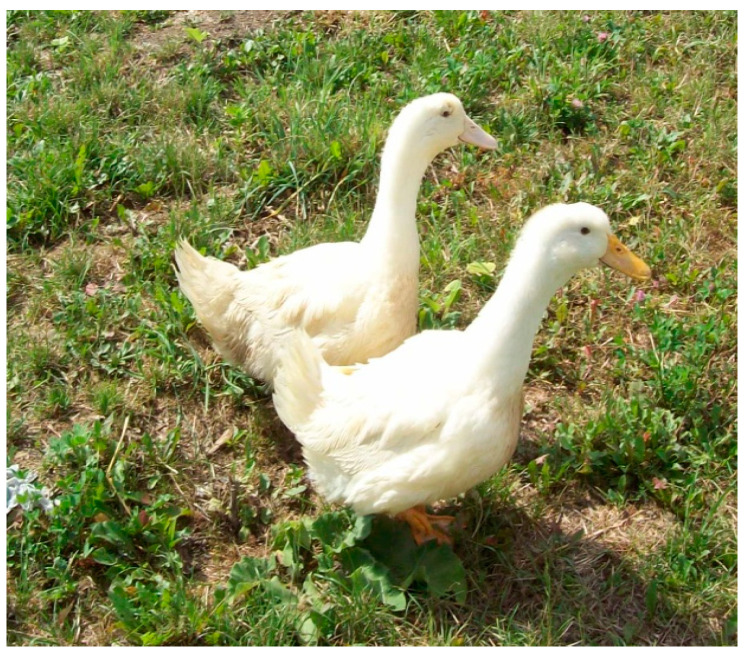
Male and female ducks from the P33 strain.

**Figure 2:**
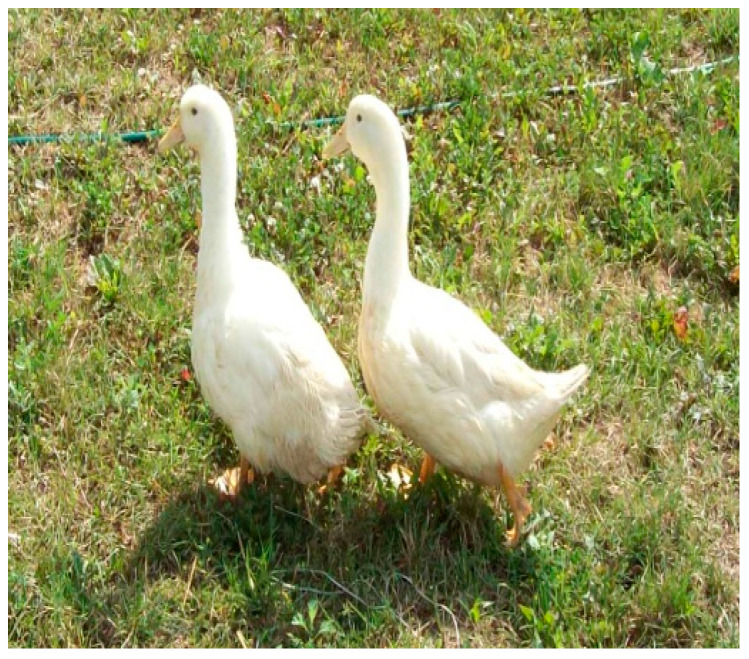
Male and female ducks from the P8 strain.

**Figure 3:**
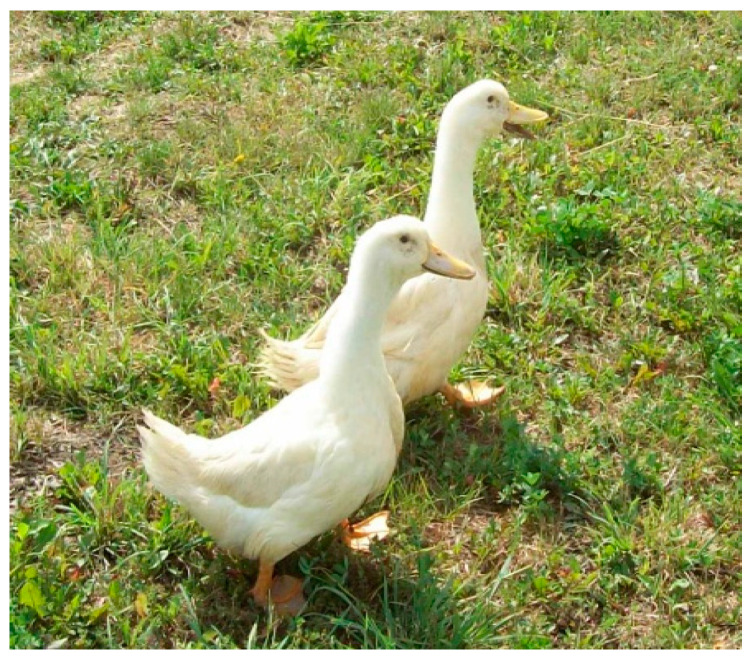
Male and female ducks from the P9 strain.

Ducks from the P33 (Polish Pekin), P8 (Danish Pekin), and P9 (French Pekin) strains, included in the programme for the conservation of genetic resources in Poland [26], were used for the study (Figure 1, Figure 2 and Figure 3). One-day-old, sexed ducklings were obtained from Waterfowl Genetic Resources Station in Dworzyska near Kórnik, belonging to the National Research Institute of Animal Production in Kraków, Poland. Ducks were collated by strain, in 3 groups of 60 birds each. Two subgroups were distinguished in each group, comprising 30 males and 30 females. A total of 180 birds were used in the experiment. Throughout the experiment (49 days), the ducks were housed in a conventional poultry building under the same environmental conditions where incandescent lighting was used. Infrared heaters were used up to and including day 21. The ducks were kept on straw in six pens. Each 12 m^2^ pen housed 30 birds.

During the rearing period (1–49 d), ducks from the compared strains were fed the same industrial complete feed mixtures with the same ingredients and chemical composition. From the 1st to the 21st day of life, ducks were fed an industrial starter complete feed mixture for waterfowl poultry in the form of crumbles containing 20.7% CP (crude protein) and 12.5 MJ (2996 kcal) metabolizable energy (ME) in 1 kg of feed and, from the 22nd to the 49th day of rearing, a grower/finisher feed mixture in the form of pellets containing 17.5% CP and 12.5 MJ (2985 kcal) ME. The basic chemical composition of the feed mixtures used was determined at the Biological and Chemical Laboratory of Bydgoszcz University of Science and Technology, Poland, and the results of the analysis are presented in Table 1. The ingredient composition of the complete industrial feed mixtures used in duck feeding was presented in an earlier paper [27]. Throughout the rearing period (1–49 d), the ducks were provided with 24 h unlimited access to water.

### 2.2. Obtaining Meat Samples

At 49 days of age, seven males and seven females were selected for dissection from each strain (42 birds in total) of the genetic resource ducks being compared. The birds with the body weight closest to the arithmetic mean value of the body weight of males or females of the strain were selected for slaughter. The average body weights of 49-day-old males selected for dissection were 2055 ± 56.1 g (strain P33, Polish Pekin), 2193 ± 125.9 g (strain P8, Danish Pekin), and 1870 ± 137.1 g (strain P9, French Pekin), while those of females were 1960 ± 49.2 g (strain P33), 2113 ± 136.1 g (strain P8), and 2088 ± 36.9 g (strain P9). The standard deviation (sd) of BW of the entire population of the three duck strains evaluated was ± 134.5 g. The body weight of the birds at 49 days old was determined individually using an electronic hook scale.

**Figure 4:**
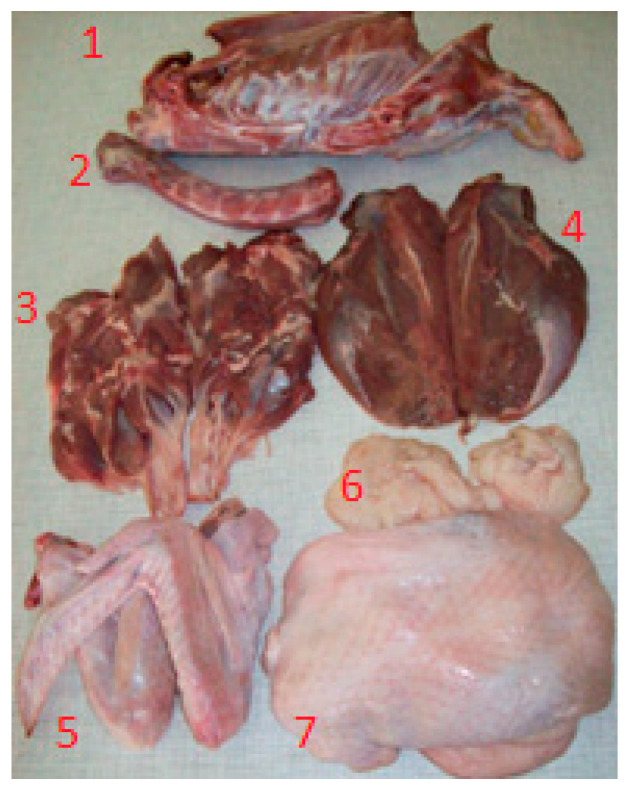
Carcass parts extracted during dissection. Explanation: 1—carcass remainders; 2—neck without skin; 3—leg muscles; 4—breast muscles; 5—wings; 6—abdominal fat; 7—skin with subcutaneous fat.

Feeding of ducks was stopped 12 h before slaughter. Until the birds were caught for slaughter, they were provided with unlimited access to water. Manual slaughter (stunning before hitting the head with a baton, cutting the neck vessels, bleeding), scalding, feather removal, and evisceration of the ducks were performed on the experimental farm. Eviscerated carcasses with necks were cooled in a Hendi refrigerated cabinet (Hendi, Gądki, Poland) at 4 °C for 18 h. The cooled carcasses were weighed on an electronic balance WLC 6/12/F1/R (Radwag, Radom, Poland) with an accuracy of 0.1 g. Once the weight of the carcasses was determined, carcass dissection was performed according to the method given by Ziołecki and Doruchowski [28] (Figure 4).

After dissection of the duck carcasses, the breast muscles (m. pectoralis major) and leg muscles (thigh muscle) were sampled to determine the fatty acid profile and sensory characteristics.

### 2.3. Fatty Acid Profile

The content of individual fatty acids in the lipids of the breast and leg muscles of the evaluated groups of ducks was determined at the Biological and Chemical Laboratory of the Faculty of Animal Breeding and Biology, Bydgoszcz University of Science and Technology. The collected samples were lyophilised in an Alpha 1–4 LD plus lyophiliser (Christ, Osterode, Germany). Fat extraction (by shaking) was then performed using an extraction mixture of chloroform: methanol for 1 h, fat methylation with 0.5 M sodium methanol at 37 °C for 22 h. Isooctane was then introduced to extract fatty acid esters. Chromatographic separation of fatty acid methyl esters was performed using a gas chromatograph (Agilent Technologies, Santa Clara, CA, USA) with a mass detector (MSD 5977A), using a 60 m × 0.25 mm × 0.25 μm HP-88 column. The temperature of the dispenser and transfer line was 250 °C; the ion source, 230 °C; the quadrupole, 180 °C; and the helium flow rate, 1.2 mL/min. Using Supelco 37 component FAME MIX formulas (Merck KGaA, Darmstadt, Germany), fatty acid methyl esters were identified in the meat samples. FA determinations were performed according to Polish standards [29]. Semi-quantitive methods were used to calculate the structure of FA. Using the FA profile data, six nutritional parameters of lipids in duck breast or leg muscle were calculated. These indices were calculated according to the formulas:

Nutritive Value Index = (C18:0 + C18:1/C16:0)—Chen et al. [30]
Atherogenic Index = (C12:0 + 4 × C14:0 + C16:0/∑UFA)—Ulbricht and Southgate [31]
Thrombogenic Index = (C14:0 + C16:0 + C18:0)/(0.5 × MUFA) + (0.5 × ∑n-6PUFA) + (3 × ∑n-3 PUFA) + (∑n-3 PUFA/∑n-6 PUFA)—Ulbricht and Southgate [31]
Health-Promoting Index = (∑UFA/4 × C14:0 + C16:0)—Chen and Liu [32]
Peroxidisability Index = (percentage of monoenoic acid × 0.025) + (percentage of dienoic acid × 1) + (percentage of trienoic acid × 2) + (percentage of tetraenoic acid × 4) + (percentage of pentaenoic acid × 4) + (percentage of hexaenoic acid × 8)—Ericson [33]
Hypocholesterolemic/hypercholesterolemic ratio (H/H) = (C18:1 + ∑PUFA/(C14:0 + C16:0)—Chen and Liu [32].

In addition, the PUFAn6 and PUFAn3 contents and PUFA n6/n3 ratio were calculated.

### 2.4. Sensory Evaluation

The evaluation of sensory properties was carried out on heat-treated meat obtained from the breast (m. pectoralis major) and legs (thigh muscle) of birds slaughtered at 49 days of age. Juiciness, tenderness, as well as the intensity and desirability of the meat’s taste and aroma were determined during the evaluation. Heat treatment of breast or leg meat samples was conducted in a 0.6% brine solution. An amount of 200 mL of water was added per 100 g of meat. The samples were treated until reaching a temperature of 80 °C. After thermal treatment, the samples were cooled to 60 °C and subjected to sensory assessment [34]. The evaluation of the sensory properties of the meat was performed by a panel of 6 trained judges, according to a scale provided by Baryłko-Pikielna and Matuszewska [35]. A 5-point grading scale determined the intensity of aroma and taste (1 pt.—undetectable, 2 pts.—perceptible, 3 pts.—weakly decisive, 4 pts.—strong, 5 pts.—very strong), desirability of the aroma and taste (1 pt.—very undesirable, 2 pts.—slightly undesirable, 3 pts.—indifferent, 4 pts.—desirable, 5 pts.—highly desirable), juiciness of the meat (1 pt.—markedly dry, 2 pts.—dry, 3 pts.—poorly succulent, 4 pts.—juicy, 5 pts.—very juicy), and tenderness (1 pt.—meat very firm, 2 pts.—tough, 3 pts.—slightly tender, 4 pts.—tender, 5 pts.—very tender).

### 2.5. Statistical Analysis

The collected numerical data on the FA profile, health lipid indices, and sensory traits of the compared duck strains—P33, P8, and P9—included in the genetic resource conservation programme were subjected to statistical analysis. Arithmetic means and standard error of the mean (SEM) (total for the three strains) were calculated for each trait evaluated. In the next stage, the Shapiro–Wilk test was used to assess the conformity of the empirical distributions of the traits studied to a normal distribution. In this research, a two-factor analysis of variance was used to determine the effect of strain (genotype) and sex of ducks on the evaluated traits of duck meat quality. The following linear model was used: Yijk = µ + ai + bj + (a · b)ij + eijk, where Yijk is the effect of the value of the analysed trait, µ is the overall mean for the tested trait, ai is the effect of i-th strain of ducks, bj is the effect of j-th sex of ducks, (a · b)ij is the effect of genotype by sex interaction, eijk is a random error. Statistical characteristics of the traits were performed using the SAS computer program, version 9.4 [36]. The significance of differences (*p* < 0.05) between the strains was verified using Tukey’s post hoc test. For all meat quality traits studied, the individual bird (meat sample) was the experimental unit.

## 3. Results

The compared duck strains differed significantly (*p* < 0.05) in the content of myristic (C14:0), palmitic (C16:0), oleiec (C18:1n9), linoleic (C18:2n6), and arachidonic (C20:4n6) acids (Table 2). The breast muscles of the P8 and P9 ducks of both sexes were characterised by a significantly (*p* < 0.05) higher content of C14:0 and C18:1n9 acids compared to the muscles of P33 ducks. The C16:0 content of the breast muscle lipids of P9 ducks was significantly higher than that of P33 and P8 ducks, while the C20:4n6 acid content of the breast muscles of 49-day-old P8 ducks was significantly lower than that of P33 ducks. P8 ducks had a significantly higher C18:2n6 content in breast muscles compared to P33 and P9 ducks (Table 2). Regardless of origin, males had significantly (*p* < 0.05) higher C18:1n9 content in breast muscles compared to those of females. In contrast, the breast muscles of females had a significantly (*p* < 0.05) higher C22:6n3 content than those obtained from males. The interaction of genotype versus sex was significant for C14:0, C16:0, C16:1, C18:1n9, C18:2n6, and C20:4n6 contents.

The results of fatty acid profile determinations in the lipids of leg muscles of 49-day-old ducks, however, indicate a significant (*p* = 0.017) effect of the genotype on C16:0 content (Table 3). Leg muscles of P9 ducks were significantly inferior to those of P33 and P8 ducks in terms of the percentage of C16:0 acid content. The sex of the birds had no significant (*p* > 0.05) effect on the fatty acid profile of leg muscle lipids of 49-day-old Pekin ducks from the compared strains. The interaction of genotype versus sex was significant for C14:0 (*p* = 0.012) and C16:0 (*p* = 0.024) contents.

There were more saturated fatty acids (SFAs) than unsaturated fatty acids (UFAs) in the lipids of breast and leg muscles. Leg muscle lipids contained more UFA than breast muscle lipids (Table 4 and Table 5). The origin of the ducks had a significant effect on the percentage of monounsaturated fatty acids (MUFAs) in the breast muscles and the proportion (%) of MUFA and UFA in the leg muscles. The breast muscles of P33 ducks contained significantly (*p* < 0.05) less MUFA than the breast muscles of P8 and P9 ducks. In contrast, the leg muscles of P8 ducks contained significantly (*p* < 0.05) less MUFA than the leg muscles of P33 and P9 ducks. The leg muscles of P8 ducks also contained significantly less UFA than those of P9 birds. The duck genotype had a significant effect on the PI of the breast muscles and the Nutritive Value Index and Health-Promoting Index of the leg muscles. P33 ducks were characterized by a significantly (*p* = 0.041) higher PI of breast muscles than the PI values of the breast meat of ducks from the P8 and P9 strains (Table 4) The Nutritive Value Index and Health-Promoting Index of the P9 duck leg muscles were significantly higher (*p* = 0.003 and *p* = 0.041) than the P33 and P8 duck leg muscles (Table 5). The sex of the birds interacted significantly (*p* < 0.05) with the MUFA, PUFAn6, PUFAn3, and PI contents of the breast muscles. The interaction of genotype vs. sex was significant for the PUFA n6 (*p* = 0.025) and PI (*p* = 0.008) of breast muscles and the PUFAn6 of leg muscles (*p* = 0.038) of the ducks tested at 49 days of age.

The results of the evaluation of the sensory characteristics of the breast and leg muscles obtained after slaughtering 49-day-old Pekin ducks of the P33, P8, and P9 strains are summarised in Table 6 and Table 7. The breast muscles of P8 ducks of both sexes after heat treatment were statistically different *(p* < 0.05) and characterised by lower aroma intensity compared with those of P33 ducks and significantly lower aroma desirability than the breast muscles of P33 and P9 ducks. The breast meat of P33 ducks had significantly higher juiciness compared to the breast muscles of P8 and P9 ducks.

The genotype of the birds had a significant effect (*p* < 0.05) on the sensory properties of leg muscles (Table 7). The leg muscles of the P33 ducks had significantly (*p* < 0.05) worse tenderness than the leg muscles of the P8 and P9 ducks. In addition, significant (*p* < 0.05) differences were found between males and females for the aroma and taste desirability of breast muscles (*p* = 0.012 and *p* = 0.002) and the aroma intensity of leg muscles (*p* = 0.036). The interaction of genotype versus sex was significant for the aroma intensity and aroma desirability of leg muscles. 

## 4. Discussion

For the discussed traits’ FA profiles, health lipid indices, and sensory traits, individual bird (meat sample) was the experimental unit. Therefore, in the presented arrangement for the presentation of the results of our own research, *n* = 14 for strain, while for sex, *n* = 21 for all the mentioned meat quality traits of the 49-day-old ducks from the P33, P8, and P9 strains.

Myristic acid (C14:0) and palmitic acid (C16:0), which adversely affect human health, were found in the lipids of the breast and leg muscles of the ducks studied [37]. However, the proportion of myristic acid in breast muscle lipids was lower than in pedigree ducks [38], hybrid ducks [39], and mallard ducks [40]. In an experiment by Kokoszyński and Bernacki [7], the contents of myristic acid in the breast muscles of 49-day-old P11 and P22 ducks were slightly lower than in the evaluated P33, P8, and P9 ducks. On the other hand, Witak [9] found a lower content of C14:0 acid (0.33%) in the leg muscles of 49-day-old A44 ducks. Compared to the ducks evaluated, lower amounts of myristic acid in duck leg muscles were also obtained by Muhlisin et al. [8] in native Korean ducks (0.55%) and imported commercial ducks (0.61%), while higher C14:0 contents (0.95–1.03%) were observed Bombik et al. [40] in the leg muscles of Mallard ducks (*Anas platyrhynchos* L.). The content of stearic acid (which is considered biologically neutral for human health—[37]) in the breast and leg muscles of the ducks evaluated was significantly higher than in the breast muscles (5.57%) and leg muscles (4.89%) of A44 ducks aged 49 days [9] and in the pectoral muscles of 49-day-old hybrids (6.97 to 7.33%) in an experiment of Heo et al. [39]. Compared to the results obtained in our study, a similar C18:0 content (17.4%) was found in the breast muscles of 8-week-old native Korean ducks [41] and the leg muscles of 49-day-old P33, K2, and A3 ducks (12.74, 13.78, and 10.69%, respectively) [42]. In contrast, Kokoszyński and Bernacki [7] found a higher C18:0 content in the leg muscles of P22 ducks (20.96%). The FA profile is determined by many factors. As we mentioned in the Section 1, these factors include, among others, the bird species, diet composition, type of muscle, housing system, and the age and sex of the bird. The different FA profiles of meat lipids may also result from the analytical method used for its determination. In beef studies, De Smet et al. [43] indicated that, as carcass fatness increases, the contents of SFA and MUFA increase faster than PUFA. 

Analysing the ducks evaluated in terms of the profile of UFA contained in the breast and leg muscles, it can be concluded that their muscles were characterised by lower contents of C16:1, C18:1, and eicosine C20:1 acids compared to the breast and leg muscles of the 49-day-old pedigree ducks evaluated by Wołoszyn et al. [38] and Witak [9]. Breast muscles of the evaluated genetic resources ducks at 49 days of age were characterised by a similar C18:2 content compared to the breast meat from the strain ducks evaluated by Kokoszyński and Bernacki [7] and Wołoszyn et al. [38]. In contrast, in the same experiments, a lower C18:2 content was found in duck leg muscles than in the P33, P8, and P9 birds evaluated. In contrast, Kim et al. [41] and Kwon et al. [44] obtained significantly higher C18:2 contents in the breast and leg muscles of 56-day-old native Korean ducks and imported commercial ducks at 42 days of age. The content of C20:4 in the breast muscle lipids of the assessed ducks was higher than that assessed by Heo et al. [33] and similar to the results of Wołoszyn et al. [38]. The breast and leg muscles of the P33, P8, and P9 ducks assessed also contained C22:6. However, its content in the breast and leg muscles was lower than in the breast and leg muscles of ducks of the same genotype as assessed by Wołoszyn et al. [42]. There were more SFA than UFA in the breast and leg muscles of the duck strains compared. This may adversely affect the nutritional value of the meat of the ducks studied. In an experiment by Witak [9], the proportion of SFA in breast and leg muscles from 49-day-old A44 breeding ducks did not exceed 25%. In contrast, Kokoszyński and Bernacki [7] found a higher proportion of SFA in the breast and leg muscles of P11 (45.61 and 38.28%, respectively) and P22 (48.06 and 47.33%) ducks at 49 days of age. Leskanich and Noble [1], reporting the FA composition of different poultry species, showed a higher SFA content in duck meat (50.3%) compared to broiler chicken meat (42.0%). The results of a study by Wołoszyn et al. [38] indicate a lower proportion of SFA in the breast muscles of pedigree ducks, A55 (34.17%) and P66 (34.53%), than in the breast muscles of genetic resource ducks from the P33 (42.04%), K2 (38.84%), and A3 (38.16%) strains. In relation to leg muscles, a higher content of SFA was found in P-33, K2, A3, and SB than in A55 and P66 pedigree ducks [39]. The proportion of MUFA in the breast muscle lipids was lower than in the leg muscle lipids of the ducks studied. A higher proportion of MUFA in the pectoral and leg muscles of ducks was found, among others, by Kokoszyński and Bernacki [7], Muhlisin et al. [8], and, especially, Heo et al. [39]. In Witak’s study [9], as in the present assessment, the proportion of MUFA in leg muscles was higher than in pectoral muscles. In contrast, the proportion of PUFA in the lipids of the breast and leg muscles of the compared duck strains was greater than in the muscles of Pekin ducks in assessments by Smith et al. [45] and Leskanich and Noble [1] (17.0 and 16.5%, respectively). In a study by Witak [9], the PUFA acid content of the breast muscles of 49-day-old A44 ducks was only 7.16%, lower than that of the breast muscles of the ducks evaluated. In another experiment [44], the breast muscles of 56-day-old native Korean ducks contained 39.0% PUFA, while those of imported commercial ducks at 6 weeks of age contained 35.0% PUFA, which was more than that of the evaluated P33, P8, and P9 ducks. The proportion of PUFA in leg muscle lipids was similar to the PUFA content in the breast muscles of the compared duck strains. In studies by Kokoszyński and Bernacki [7] and Witak [9], the proportion of PUFA in leg muscles was lower than in the evaluated ducks. Wołoszyn et al. [46] also found a higher proportion of PUFA in the leg muscles of P33, K2, A3, and SB genetic reserve ducks than in the muscles of A55 and P66 breeding ducks. In contrast, the proportion of UFA in the lipids of the breast and leg muscles of the 49-day-old P33, P8, and P9 ducks assessed was similar to that determined in the muscles of A44 breeding ducks of the same age [9]. A higher proportion of UFA in the breast and leg muscles of ducks was obtained by, among others, Heo et al. [39], Kokoszyński and Bernacki [7], Muhlisin et al. [8], and Wołoszyn et al. [38,42].

The obtained differences between the FA profile of the breast and leg muscles of the compared duck strains and between the FA profile of the lipids of the breast and leg muscles (thighs) were probably related to the different fat contents in the meat [47]. Additionally, the different types of fibres and their cellular metabolism may have influenced the differentiation of the FA composition of muscle [48]. The obtained results were also affected by the different susceptibilities of PUFA to lipid oxidation processes during the storage of meat prior to the determination of the FA profile.

The ratio of UFA to SFA in the lipids of the breast and leg muscles of the ducks studied ranged from 0.9 to 1.0 and was lower, i.e., less favourable, than in studies by Kokoszyński and Bernacki [7], Witak [9] and Wołoszyn et al. [38], in which it exceeded a value of 1.8. The PUFA/SFA ratio in muscles obtained from 7-week-old ducks in our study ranged from 0.4 to 0.5 and was lower than that calculated for duck muscles in studies by Kim et al. [33] and Wołoszyn et al. [32]. Larger PUFA/SFA values were found in the breast muscles of Pekin ducks by Witak [9], while Bombik et al. [40] found them in the breast muscles of Mallard ducks. The PUFA/SFA ratio in the lipids of the leg muscles of the ducks studied was similar to the PUFA/SFA ratio in the muscles from the legs of P11 (0.51) and P22 (0.42) ducks assessed by Kokoszyński and Bernacki [7]. A similar value of the PUFA/SFA ratio in the pectoral muscles of 49-day-old P33, K2, and A3 ducks (0.74–0.92) was obtained by Wołoszyn et al. [49], while Witak [9] obtained higher values in this ratio (1.54) for the breast muscles of A44 pedigree ducks at 7 weeks of age.

In addition, our study found significant (*p* < 0.05) differentiation between males and females in terms of C18:1n9 and C22:6n3 and MUFA, PUFAn6, and PUFAn3 contents in pectoral muscles. Kokoszynski [14], comparing meat traits of commercial hybrid Pekin ducks, found statistically significant differences between male and female PP45 ducks in terms of C16:1, C18:0, C18:1, and C20:4 acid contents in pectoral muscles and in terms of the percentage of C20:4 in leg muscle lipids. In the same experiment [14], statistically significant differences were found between male and female Star 53 HY ducks in terms of C18:0 and C18:1 contents in leg muscles.

The compared duck strains were characterised by a higher value of AI of breast muscles (AI = 1.1) and a desirable AI of leg muscles (AI = 0.8) in relation to the value recommended in terms of human health (AI < 1.0)—Wereńska et al. [50]. However, in relation to both types of muscles, higher TI values (TI > 1.9) were found in relation to the nutritional recommendations (TI < 0.5). It is widely believed that the consumption of food products with a low value of AI and TI should better protect the human body against potential coronary artery disease [51]. 

The results of sensory evaluation of the breast muscles of the studied duck strains indicate that they differ slightly in terms of aroma (intensity and desirability) and juiciness. Kokoszyński [14], conducting a study on utility hybrids of Pekin ducks, found that the breast muscles of Star 53 HY ducks at 42 and 49 days of age were characterised by statistically significantly higher juiciness and more intense aroma compared to those of AP54, PP45, and PP54 ducks. Chartrin et al. [3] found greater tenderness and juiciness of the breast muscles of 98-day-old Pekin ducks compared to the breast muscles of Muscovy, Hinna, and Mullard ducks of the same age. The results of a study by Wawro et al. [52] indicate greater tenderness and juiciness and better tastiness and aroma of Pekin duck breast muscles than muscles from Muscovy and mule duck breasts. In an experiment by Okruszek et al. [53], Pekin ducks (strains P8 and P33) had breast muscles with greater tenderness and juiciness compared to breast muscles from Khaki Campbell (strain Kh1), Orpington (strain O1), and mini (strain K2) ducks. In another experiment [54], significant differences were found between P9 (Pekin ducks of French origin), K2 (crosses of Pekin duck and wild mallard), and KhO1 (crosses of Khaki Campbell drakes and Orpington Fauve ducks) ducks in terms of the aroma desirability, taste intensity, and taste desirability of breast muscles and the aroma desirability of leg muscles assessed after heat treatment. In contrast, Michalczuk et al. [55], evaluating the sensory properties of the breast muscles of 49-day-old Pekin ducks of the P44 conservative strain, found lower tenderness and juiciness and lower overall consumer acceptability of meat from intensively reared ducks than meat obtained from birds kept with access to a paddock, as well as higher tenderness and overall acceptability of breast muscles of females than males, regardless of the housing system.

## 5. Conclusions

In summary, the significant differences in the evaluated FA profiles and sensory attributes may indicate the different nutritional values and culinary suitability of the meat of ducks from the P33, P8, and P9 strains included in the Genetic Resources Protection Programme. The compared duck strains differed significantly in the contents of myristic, palmitic, oleic, linolenic, and arachidonic acids in breast muscle lipids and the amount of palmitic acid in leg muscle lipids. The breast muscles of P33 ducks contained significantly less MUFA than the breast muscles of P8 and P9 ducks. Leg muscles from P8 ducks of both sexes contained a significantly lower percentage of UFAs than those from P9 ducks and significantly less (%) MUFAs than leg muscles from P33 and P9 ducks. P33 ducks were characterized by a significantly higher PI of breast muscles, while P9 ducks had higher Nutritive Value Index and Health-Promoting Index for leg muscles compared to the remaining strains of ducks assessed. Regardless of the origin of the ducks, males compared to females had significantly higher C18:1n9 and MUFA contents, PI, and aroma and taste desirability for breast muscles and aroma intensity for leg muscles. In contrast, females had higher C22:6n3, PUFAn6, and PUFAn3 contents in pectoral muscles compared to pectoral muscles obtained from males. The breast meat of P8 ducks was characterised by poorer juiciness and lower aroma intensity than the breast muscles of P33 ducks and less desirability of aroma than the breast meats of P33 and P9 ducks. P9 ducks had significantly poorer juiciness of breast muscles than P33 ducks. The leg muscles of the P33 ducks had worse tenderness than the leg muscles of the P8 and P9 ducks. The high content of PUFAs and the high sensory qualities increase the attractiveness of the meat of the evaluated duck strains as a food product.

## Figures and Tables

**Table 1:** Chemical composition of compound feeds.

Chemical Composition (% of Feed)	Feed Mixture	
	Starter	Grower/ Finisher
	Days of Life	
	1–21	22–49
Dry matter	90.8	89.2
Crude protein	20.7	17.5
N-free extracts ^A^	55.0	58.9
Crude fat	5.7	5.0
Crude fibre	4.5	3.5
Crude Ash	4.9	4.3
ME ^B^ MJ kcal of kg feed	12.5 2996	12.5 2985
MJ:1% CP kcal:1% CP	0.6 145	0.7 171

^A^ %N-free extracts = %dry matter—(% crude protein + crude fat + crude fibre + crude ash). ^B^ The values calculated from ingredient AME values.

**Table 2:** Fatty acid profile (%) of breast muscle lipids of 49-day-old Pekin ducks.

Fatty Acid ^A^	Strain (G)			Sex		SEM	*p*-Value		
	P33	P8	P9	Male	Female		G	S	G × S
C14:0 (myristic)	0.4 b	0.5 a	0.5 a	0.5	0.4	0.1	0.004	0.284	0.005
C16:0 (palmitic)	40.8 b	40.0 b	41.6 a	41.7	41.2	0.2	0.043	0.202	0.019
C16:1 (palmitoleic)	0.5	0.6	0.6	0.6	0.6	0.1	0.059	0.258	0.011
C17:0 (margaric)	0.2	0.2	0.2	0.2	0.2	0.1	0.182	0.116	0.271
C18:0 (stearic)	17.3	16.3	17.0	16.5	17.1	0.2	0.132	0.147	0.187
C18:1n9 (oleic)	13.4 b	15.0 a	14.9 a	15.3	13.9 *	0.4	0.014	0.023	0.047
C18:2n6 (linoleic)	12.7 b	15.4 a	12.2 b	12.2	14.6	0.2	0.041	0.174	0.018
C20:0 (arachidic)	0.4	0.6	0.5	0.5	0.5	0.1	0.093	0.653	0.074
C20:1 (gadoleic)	0.3	0.3	0.3	0.3	0.3	0.1	0.053	0.102	0.397
C20:2 (eicosadienoic)	0.4	0.4	0.4	0.4	0.4	0.1	0.185	0.865	0.543
C22:0 (behenic)	0.5	0.4	0.4	0.4	0.4	0.1	0.067	0.124	0.104
C20:4n6 (arachidonic)	12.3 a	9.8 b	10.7 ab	10.3	11.6	0.5	0.021	0.076	0.007
C24:1n9 (nervonic)	0.4	0.4	0.4	0.4	0.4	0.1	0.585	0.800	0.054
C22:6n3 (docosahexaenoic)	0.7	0.6	0.6	0.6	0.7 *	0.1	0.464	0.046	0.098

^A^ Common names are given in parentheses. Note: *n* = 14 for strain; *n* = 21 for sex. Values in rows marked with different letters differ significantly between strains (*p* < 0.05). * Significant differences between males and females (*p* < 0.05).

**Table 3:** Fatty acid profile (%) of leg muscle lipids of 49-day-old Pekin ducks.

Fatty Acid ^A^	Strain (G)			Sex (S)		SEM	*p*-Value		
	P33	P8	P9	Male	Female		G	S	G × S
C14:0 (myristic)	0.7	0.7	0.7	0.7	0.7	0.1	0.332	0.564	0.012
C16:0 (palmitic)	37.6 a	37.3 a	36.1 b	37.1	36.7	0.2	0.017	0.721	0.024
C16:1 (palmitoleic)	1.6	1.5	1.7	1.5	1.6	0.1	0.565	0.530	0.152
C17:0 (margaric)	0.2	0.2	0.2	0.2	0.2	0.1	0.095	0.440	0.760
C18:0 (stearic)	12.1	12.9	12.8	12.6	12.6	0.3	0.349	0.927	0.107
C18:1n9 (oleic)	22.5	21.6	22.8	22.3	22.3	0.3	0.128	0.790	0.164
C18:2n6 (linoleic)	19.3	18.6	18.6	18.9	18.9	0.2	0.291	0.972	0.775
C20:0 (arachidic)	1.4	1.3	1.3	1.3	1.4	0.1	0.579	0.676	0.597
C20:1 (gadoleic)	0.4	0.4	0.4	0.4	0.4	0.1	0.110	0.487	0.266
C20:2 (eicosadienoic)	0.2	0.2	0.2	0.2	0.2	0.1	0.477	0.496	0.588
C22:0 (behenic)	0.2	0.2	0.2	0.2	0.2	0.1	0.059	0.410	0.363
C20:4n6 (arachidonic)	3.6	4.7	4.6	4.3	4.0	0.2	0.068	0.053	0.154
C24:1n9 (nervonic)	0.2	0.2	0.2	0.2	0.2	0.1	0.054	0.147	0.125
C22:6n3 (docosahexaenoic)	0.2	0.3	0.3	0.3	0.2	0.1	0.055	0.554	0.202

^A^ Common names are given in parentheses. Note: *n* = 14 for strain; *n* = 21 for sex. Values in rows marked with different letters differ significantly between strains (*p* < 0.05).

**Table 4:** Fatty acid balance of breast muscle lipids of 49-day-old Pekin ducks.

Item	Strain (G)			Sex (S)		SEM	*p*-Value		
	P33	P8	P9	Male	Female		G	S	G × S
Saturated fatty acids (SFAs), %	59.6	60.1	60.0	59.9	59.8	0.3	0.756	0.890	0.160
Monounsaturated fatty acids (MUFAs), %	14.6 b	16.7 a	16.1 a	16.6	15.0 *	0.1	0.049	0.047	0.056
Polyunsaturated fatty acids (PUFAs), %	25.9	23.2	24.0	23.5	25.1	0.2	0.613	0.914	0.146
Unsaturated fatty acids (UFAs), %	40.1	39.9	40.0	39.9	40.2	0.3	0.727	0.934	0.181
UFA/SFA ratio	0.7	0.7	0.6	0.7	0.7	0.1	0.313	0.364	0.372
PUFA/SFA ratio	0.5	0.4	0.4	0.4	0.4	0.1	0.081	0.990	0.336
PUFA n6	25.0 a	25.2 a	22.9 b	22.5	26.2 *	0.3	0.002	0.026	0.025
PUFA n3	0.7	0.6	0.6	0.6	0.7 *	0.1	0.464	0.046	0.098
n6/n3	35.7	42.0	38.2	37.5	37.4	0.1	0.770	0.809	0.266
Nutritive Value Index	0.7	0.7	0.8	0.7	0.7	0.1	0.111	0.797	0.071
Atherogenic Index	1.1	1.1	1.1	1.1	1.1	0.1	0.215	0.703	0.065
Thrombogenic Index	2.6	2.7	2.8	2.7	2.6	0.3	0.143	0.671	0.052
Health-Promoting Index	0.9	1.0	0.9	0.9	0.9	0.1	0.077	0.644	0.088
Peroxidisability Index	68.3 a	59.4 b	60.6 b	65.5	59.3 *	2.7	0.041	0.036	0.008
HH index	1.0	0.9	0.9	0.9	0.9	0.1	0.923	0.370	0.067

Note: *n* = 14 for strain; *n* = 21 for sex. HH index—hypocholesterolemic/hypercholesterolemic ratio Values in rows marked with different letters, differ significantly between strains (*p* < 0.05). * Significant differences between males and females (*p* < 0.05).

**Table 5:** Fatty acid balance of leg muscle lipids of 49-day-old Pekin ducks.

Item	Strain					SEM	*p*-Value		
	P33	P8	P9	Male	Female		G	S	G × S
Saturated fatty acids (SFAs), %	52.1	52.6	51.3	52.0	51.9	0.2	0.054	0.802	0.359
Monounsaturated fatty acids (MUFAs), %	24.5 a	23.6 b	25.0 a	24.3	24.5	0.1	0.024	0.851	0.452
Polyunsaturated fatty acids (PUFAs), %	23.4	23.8	23.7	23.7	23.6	0.2	0.129	0.874	0.497
Unsaturated fatty acids (UFAs), %	47.9 ab	47.4 b	48.7 a	48.0	48.1	0.2	0.033	0.872	0.414
UFA/SFA ratio	0.9	0.9	1.0	0.9	0.9	0.1	0.313	0.364	0.372
PUFA/SFA ratio	0.5	0.5	0.5	0.5	0.5	0.1	0.081	0.990	0.336
PUFAn6	22.9	23.2	23.2	23.2	22.9	0.3	0.703	0.830	0.038
PUFAn3	0.2	0.3	0.3	0.3	0.2	0.1	0.056	0.554	0.202
n6/n3	114.5	77.3	87.3	97.3	114.5	0.1	0.105	0.314	0.164
Nutritive Value Index	0.9 b	0.9 b	1.0 a	0,9	0.9	0.1	0.003	0.658	0.096
Atherogenic Index	0.8	0.8	0.8	0.8	0.8	0.1	0.073	0.596	0.105
Thrombogenic Index	2.1	2.1	2.0	2.0	2.1	1.7	0.241	0.265	0.260
Health-Promoting Index	1.2 b	1.2 b	1.3 a	1.2	1.2	0.1	0.041	0.684	0.051
Peroxidisability Index	35.1	40.6	40.2	38.7	39.3	0.1	0.115	0.729	0.065
HH index	1.2	1.3	1.3	1.3	1.3	0.1	0.923	0.370	0.067

Note: *n* = 14 for strain; *n* = 21 for sex. HH index—hypocholesterolemic/hypercholesterolemic ratio. Values in rows marked with different letters differ significantly between strains (*p* < 0.05).

**Table 6:** Sensory characteristics of the breast muscles of 49-day-old Pekin ducks.

Item	Strain (G)			Sex (S)		SEM	*p*-Value		
	P33	P8	P9	Male	Female		G	S	G × S
Aroma intensity, pts.	4.4 a	4.0 b	4.2 ab	4.3	4.0	0.1	0.028	0.068	0.694
Aroma desirability, pts.	4.5 a	4.1 b	4.4 a	4.4	4.2 *	0.1	0.006	0.012	0.730
Juiciness, pts.	4.7 a	4.5 b	4.4 b	4.6	4.4	0.1	0.016	0.096	0.149
Tenderness, pts.	4.6	4.5	4.4	4.4	4.4	0.1	0.257	0.603	0.101
Taste intensity, pts.	4.4	4.3	4.3	4.4	4.2	0.1	0.992	0.091	0.132
Taste desirability, pts.	4.4	4.3	4.4	4.5	4.2 *	0.1	0.307	0.002	0.263

Values in rows marked with different letters differ significantly between strains (*p* < 0.05). * Significant differences between males and females (*p* < 0.05).

**Table 7:** Sensory characteristics of the leg muscles of 49-day-old Pekin ducks.

Item	Strain (G)			Sex (S)		SEM	*p*-Value		
	P33	P8	P9	Male	Female		G	S	G × S
Aroma intensity, pts.	4.2	4.2	4.2	4.3	4.0	0.1	0.787	0.036	0.009
Aroma desirability, pts.	4.3	4.4	4.3	4.4	4.2 *	0.1	0.457	0.170	0.041
Juiciness, pts.	4.3	4.4	4.4	4.6	4.4	0.1	0.168	0.129	0.256
Tenderness, pts.	4.1 b	4.3 a	4.4 a	4.4	4.4	0.1	0.009	0.122	0.522
Taste intensity, pts.	4.3	4.4	4.3	4.4	4.3	0.1	0.215	0.218	0.178
Taste desirability, pts.	4.3	4.4	4.3	4.5	4.2 *	0.1	0.074	0.122	0.253

Values in rows marked with different letters differ significantly between strains (*p* < 0.05). Significant differences between males and females (*p* < 0.05).

## Data Availability

Not applicable.

## References

[1] Leskanich C.O., Noble R.C. (1997). Manipulation of the n-3 polyunsaturated fatty acid composition of avian eggs and meat. Worlds Poult. Sci. J..

[2] Batura J., Korzeniowski W., Bochno R. (1990). Effect of duck limited feeding on fatty acids composition of the deposited and muscle fats. Prz. Nauk. Lit. Zoot..

[3] Chartrin P., Méteau K., Juin H., Bernadet M.D., Guy G., Larzul C., Rémignon H., Mourot J., Duclos M.J., Baéza E. (2006). Effects of intermuscular fat levels on sensory characteristics of duck breast meat. Poult. Sci..

[4] Zanusso J., Rémignon H., Guy G., Mense H., Babilé R. (2003). The effects of overfeeding on myofibre characteristics and metabolical traits of the breast muscle in Muscovy ducks (*Cairina moschata*). Reprod. Nutr. Dev..

[5] Kowalska E., Kucharska-Gaca J., Kuźnuacka J., Biesek J., Banaszak M., Adamski M. (2020). Effects of legume-diet and sex of ducks on the growth performance, physiochemical traits of meat and fatty acid composition in fat. Sci. Rep..

[6] Huang L., Guo Q., Wu Y., Jiang Y., Bai H., Wang Z., Chen G., Chang G. (2022). Carcass traits, proximate composition, amino acid and fatty acid profiles, and minerals content of meat from Cherry Valley, Chinese crested, and crossbred ducks. Anim. Biotechnol..

[7] Kokoszyński D., Bernacki Z. (2010). Comparison of some meat traits in ducks from two conservative flocks. Arch. Tierz..

[8] Muhlisin M., Kim D.S., Song Y.R., Kim H.R., Kwon H.J., An B.K., Kang C.W., Kim H.K., Lee S.K. (2013). Comparison of meat characteristics between Korean native duck and imported commercial duck raised under identical rearing and feeding condition. Korean J. Food Sci. Anim. Resour..

[9] Witak B. (2008). Tissue composition of carcass, meat quality and fatty acid content of ducks of commercial breeding line at different age. Arch. Tierz..

[10] Onbaşilar E., Yalcin S. (2018). Fattening performance and meat quality of Pekin ducks under different rearing systems. Worlds Poult. Sci. J..

[11] Starčević M., Mahmutović H., Glamočlija N., Baltič B., Popović M., Mitrović R., Marković R., Janjić J., Glišić M., Baltić M.Ž. (2021). Pekin duck strain and housing system affect chemical composition, fatty acid profile, and the extent of lipid and protein oxidation in meat. Res. Squ.

[12] Inayat M., Abbas F., Rehman M.H., Mahmud A. (2023). Physico-chemical parameters, oxidative stress, and fatty acid profile of American Pekin ducks (*Anas platyrhynchos* domesticus) raised under different production systems. Braz. J. Poult. Sci..

[13] Cao Z., Gao W., Zhan Y., Huo W., Weng K., Zhang Y., Li B., Chen G., Xu Q. (2021). Effect of marketable age on proximate composition and nutritional profile of breast meat from Cherry Valley broiler ducks. Poult. Sci..

[14] Kokoszyński D. (2011). Evaluation of Meat Traits in Commercial Crossbreds of Pekin Type Ducks.

[15] Jarosz M., Rychlik E., Stoś K., Charzewska J. (2003). Nutrition Standards for the Polish Population and Their Importance.

[16] Chang L., Tang Q., Zhang R., Fu S., Mu C., Shen X., Bu Z. (2023). Evaluation of Meat Quality of Local Pigeon Varieties in China. Animals.

[17] Czerny M., Christlbauer M., Christlbauer M., Fischer A., Granvogl M., Hammer M., Hartl C., Hernandez N.M., Schieberle P. (2008). Re-investigation on odour thresholds of key food aroma compounds and development of an aroma language based on odour qualities of defined aqueous odorant solutions. Eur. Food Res. Technol..

[18] Wilkanowska A. (2015). Sensory evaluation of poultry meat. Poultry Farmer.

[19] Florkowski T., Słowiński M., Dasiewicz K. (2002). Colour measurements as a method for the estimation of certain chicken meat quality indicators. J. Pol. Agric. Univ. Ser. Food Sci. Technol..

[20] Gornowicz E., Dobek A., Moliński K., Szwaczkowski T. (2023). The quality of duck meat—From the perspective of physical measurements and expert judgment. Ann. Anim. Sci..

[21] Fu L., Du L., Sun Y., Fan X., Zhou C., He J., Pan D. (2022). Effect of Lentinan on Lipid Oxidation and Quality Change in Goose Meatballs during Cold Storage. Foods.

[22] Cygan-Szczegielniak D., Janicki B. (2012). Effect of age and sex of roe deer on tenderness and other quality characteristics. Food. Sci. Technol. Qual..

[23] Chaosap C., Sivapiruthep P. (2018). Meat characteristics from four different cutting parts of Cherry Valley ducks. MATEC Web Conf..

[24] Hou W., Weng K., Gu T., Zhang Y., Zhang Y., Chen G., Xu Q. (2021). Effect of muscle fiber characteristics on meat quality in fast- and slow-growing ducks. Poult. Sci..

[25] Kokoszyński D., Wilkanowska A., Saleh M., Fik M., Bigorowski B. (2021). Comparison of some meat and liver quality traits in Muscovy and Pekin ducks. J. Appl. Anim. Res..

[26] Calik J., Krawczyk K., Bielińska H., Wencek E. Program for Conservation of Genetic Resources of Duck populations. Appendix 3 to the Ordinance of the Director of the Institute of Animal Production—Polish National Research, No 48/21 from 28 December 2021. http://www.bioroznorodnosc.izoo.krakow.pl/drob/dokumenty.

[27] Kokoszyński D., Wasilewski R., Stęczny K., Kotowicz M., Hrnčár C., Arpašová H. (2019). Carcass composition and selected meat quality of Pekin ducks from genetic resources flocks. Poult. Sci..

[28] Ziołecki J., Doruchowski W. (1989). Evaluation Methods of Poultry Slaughter Value.

[29] (2021). Animal and Vegetable Fats and Oils—Gas Chromatography of Fatty Acid Methyl Esters—Part 1. Guidelines on Modern Gas Chromatography of Fatty Acid Methyl Esters.

[30] Chen Y.Y., Qiao Y., Xiao Y., Chen H., Zhao L., Huang M., Zhou H. (2016). Differences in physicochemical and nutritional properties of breast and thigh meat from crossbred chickens, commercial broilers, and spent hens. Asian-Australas. J. Anim. Sci..

[31] Ulbricht T.L.V., Southgate D.A.T. (1997). Coronary heart disease: Seven dietary factors. Lancet.

[32] Chen J., Liu H. (2020). Nutritional indices for assessing fatty acids: A mini-review. Int. J. Mol. Sci..

[33] Erickson M.C. (1992). Variation of lipid and tocopherol composition in three strains of channel catfish (*Ictalurus punctatus*). J. Sci. Food Agric..

[34] Krełowska-Kułas M. (1993). Testing of Food Products Quality.

[35] Baryłko-Pikielna N., Matuszewska I. (2009). Outline of Food Analysis.

[36] SAS Institute Inc. (2014). SAS/STAT User’s Guide.

[37] Stopler T., Mahan I.K., Escott-Stump S. (2004). Medical nutrition therapy for anemia. Krause’s Food & Nutritional Therapy.

[38] Wołoszyn J., Książkiewicz J., Skrabka-Błotnicka T., Haraf G., Biernat J., Kisiel T. (2006). Comparison of amino acid and fatty acid composition of duck breast from five flocks. Arch. Tierz..

[39] Heo K.N., Hong E.C., Kim C.D., Kim H.K., Lee M.J., Choo H.J., Choi H.C., Mushtaq M.M.H., Parvin R., Kim J.H. (2015). Growth performance, carcass yield, and quality and chemical traits of meat from commercial Korean native ducks with 2-way crossbreeding. Asian Australas. J. Anim. Sci..

[40] Bombik E., Pietrzkiewicz E., Bombik A. (2022). Analysis of the Fatty Acid Profile of the Tissues of Hunted Mallard Ducks (*Anas platyrhynchos* L.) from Poland. Animals.

[41] Kim H.K., Kang B.S., Hwangbo J., Kim C.D., Heo H.N., Choo H.J., Park D.S., Suh O.S., Hong E.C. (2012). The study on growth performance and carcass yield on meat-type Korean native ducks. Korean J. Poult. Sci..

[42] Wołoszyn J., Książkiewicz J., Orkusz A., Skrabka-Błonicka A., Biernat J., Kisiel T. (2005). Evaluation of chemical composition of ducks’s muscles from three conservative flocks. Arch. Geflügelk..

[43] De Smet S., Raes K., Demeyer D. (2004). Meat fatty acid composition as affected by fatness and genetic factors: A review. Anim. Res..

[44] Kwon H.J., Choo Y.K., Choi Y.I., Kim E.J., Kim H.K., Heo K.N., Choi H.C., Lee S.K., Kim C.J., Kim B.G. (2014). Carcass characteristics and meat quality of Korean native ducks and commercial meat-type ducks raised under same feeding and rearing conditions. Asian-Australas. J. Anim. Sci..

[45] Smith D.P., Fletcher D.L., Buhr R.J., Beyer R.S. (1993). Pekin duckling and broiler chicken pectoralis muscle structure and composition. Poult. Sci..

[46] Wołoszyn J., Książkiewicz J., Skrabka-Błotnicka T., Haraf G., Biernat J., Szukalski G. (2007). Chemical composition of leg muscles of six ducks strains. Med. Vet..

[47] Wasilewski R. (2018). Analysis of Meat Traits in Some Groups of Pekin Ducks from Genetic Resource Flocks. Ph.D. Thesis.

[48] Domínguez R., Martínez S., Carballo J., Franco I. (2004). Fatty acid profile and cholesterol and retinol contents in different locations of Celta pig breed. Grases Aceites.

[49] Wołoszyn J., Haraf G., Okruszek A., Książkiewicz J. (2011). Evaluation of duck genotype effect on some breast muscle properties. Arch. Geflügelk..

[50] Wereńska M., Haraf G., Wołoszyn J., Okruszek A., Teleszko M. (2021). Fatty acid profile and health indicies of goose meat in relation to various types of heat treatment. Poult. Sci..

[51] Fernandes C.E., Vasconcelos M.A.D.S., De Almeida Ribeiro M., Sarubbo L.A., Andrade S.A.C., Filho A.B.D.M. (2014). Natritional and lipid profiles in marine fish species from Brazil. Food Chem..

[52] Wawro K., Wilkiewicz-Wawro E., Kleczek K., Brzozowski W. (2004). Slaughter value and meat quality of Muscovy ducks, Pekin ducks and their crossbreeds, and evaluation of the heterosis effect. Arch. Tierz..

[53] Okruszek A., Wołoszyn J., Książkiewicz J., Haraf G., Szukalski G. (2006). The comparison of duck’s meat quality of different flocks. World’s Poult. Sci. J..

[54] Kokoszyński D., Bernacki Z., Biegniewska M., Saleh M., Stęczny K., Zwierzyński R., Kotowicz M., Sobczak M., Żochowska-Kujawska J., Wasilewski P.D. (2020). Carcass, physicochemical and sensort characteristics of meat from genetic reserve ducks after two reproductive seasons. S. Afr. J. Anim. Sci..

[55] Michalczuk M., Damaziak K., Pietrzak D.M., Marzec A., Chmiel M., Adamczak L., Florkowski T. (2016). Influence of housing system on selected quality characteristics of duck meat. Chapter 1. Pekin duck. Ann. Warsaw Univ. Life Sci..

